# Differences between Human Plasma and Serum Metabolite Profiles

**DOI:** 10.1371/journal.pone.0021230

**Published:** 2011-07-08

**Authors:** Zhonghao Yu, Gabi Kastenmüller, Ying He, Petra Belcredi, Gabriele Möller, Cornelia Prehn, Joaquim Mendes, Simone Wahl, Werner Roemisch-Margl, Uta Ceglarek, Alexey Polonikov, Norbert Dahmen, Holger Prokisch, Lu Xie, Yixue Li, H. -Erich Wichmann, Annette Peters, Florian Kronenberg, Karsten Suhre, Jerzy Adamski, Thomas Illig, Rui Wang-Sattler

**Affiliations:** 1 Research Unit of Molecular Epidemiology, Helmholtz Zentrum München, German Research Center for Environmental Health, Neuherberg, Germany; 2 Institute of Bioinformatics and Systems Biology, Helmholtz Zentrum München, German Research Center for Environmental Health, Neuherberg, Germany; 3 Shanghai Center for Bioinformation Technology, Shanghai, China; 4 Bioinformatics Center, Key Lab of Systems Biology, Shanghai Institutes for Biological Sciences, Chinese Academy of Sciences, Shanghai, China; 5 Institute of Epidemiology II, Helmholtz Zentrum München, German Research Center for Environmental Health, Neuherberg, Germany; 6 Institute of Experimental Genetics, Genome Analysis Center, Helmholtz Zentrum München, German Research Center for Environmental Health, Neuherberg, Germany; 7 Solid State NMR Spectroscopy and Center for Integrated Protein Science, Department Chemie, Technische Universität München, Garching, Germany; 8 Institute of Structural Biology, Helmholtz Zentrum München, German Research Center for Environmental Health, Neuherberg, Germany; 9 Institute of Laboratory Medicine, Clinical Chemistry and Molecular Diagnostics, University Hospital Leipzig, Leipzig, Germany; 10 Department of Biology, Medical Genetics and Ecology, Kursk State Medical University, Kursk, Russian Federation; 11 Department for Psychiatry, University of Mainz, Mainz, Germany; 12 Institute of Human Genetics, Helmholtz Zentrum München, German Research Center for Environmental Health, Neuherberg, Germany; 13 Institute of Epidemiology, Helmholtz Zentrum München, German Research Center for Environmental Health, Neuherberg, Germany; 14 Institute of Medical Informatics, Biometry and Epidemiology, Ludwig-Maximilians-Universität, Munich, Germany; 15 Division of Genetic Epidemiology, Department of Medical Genetics, Molecular and Clinical Pharmacology, Innsbruck Medical University, Innsbruck, Austria; 16 Faculty of Biology, Ludwig-Maximilians-Universität, Planegg-Martinsried, Germany; 17 Department of Physiology and Biophysics, Weill Cornell Medical College in Qatar, Education City - Qatar Foundation, Doha, Qatar; 18 Lehrstuhl für Experimentelle Genetik, Technische Universität München, Munich, Germany; Governmental Technical Research Centre of Finland, Finland

## Abstract

**Background:**

Human plasma and serum are widely used matrices in clinical and biological studies. However, different collecting procedures and the coagulation cascade influence concentrations of both proteins and metabolites in these matrices. The effects on metabolite concentration profiles have not been fully characterized.

**Methodology/Principal Findings:**

We analyzed the concentrations of 163 metabolites in plasma and serum samples collected simultaneously from 377 fasting individuals. To ensure data quality, 41 metabolites with low measurement stability were excluded from further analysis. In addition, plasma and corresponding serum samples from 83 individuals were re-measured in the same plates and mean correlation coefficients (*r*) of all metabolites between the duplicates were 0.83 and 0.80 in plasma and serum, respectively, indicating significantly better stability of plasma compared to serum (*p* = 0.01). Metabolite profiles from plasma and serum were clearly distinct with 104 metabolites showing significantly higher concentrations in serum. In particular, 9 metabolites showed relative concentration differences larger than 20%. Despite differences in absolute concentration between the two matrices, for most metabolites the overall correlation was high (mean *r* = 0.81±0.10), which reflects a proportional change in concentration. Furthermore, when two groups of individuals with different phenotypes were compared with each other using both matrices, more metabolites with significantly different concentrations could be identified in serum than in plasma. For example, when 51 type 2 diabetes (T2D) patients were compared with 326 non-T2D individuals, 15 more significantly different metabolites were found in serum, in addition to the 25 common to both matrices.

**Conclusions/Significance:**

Our study shows that reproducibility was good in both plasma and serum, and better in plasma. Furthermore, as long as the same blood preparation procedure is used, either matrix should generate similar results in clinical and biological studies. The higher metabolite concentrations in serum, however, make it possible to provide more sensitive results in biomarker detection.

## Introduction

Human plasma and serum are commonly used matrices in biological and clinical studies. Serum is preferred in some assays for cardiac troponins [1] whereas plasma is favored in oral glucose tolerance tests for diabetes [2]. As reviewed by Mannello [3], use of the wrong matrix (e.g. plasma in place of serum) can lead to improper diagnosis. Both plasma and serum are derived from full blood that has undergone different biochemical processes after blood collection. Serum is obtained from blood that has coagulated. Fibrin clots formed during coagulation, along with blood cells and related coagulation factors, are separated from serum by centrifugation. During this process, platelets release proteins (e.g. proinflammatory cytokines [4]) and metabolites (e.g. sphingosine-1-phosphate [5]) into the serum. To obtain plasma, an anticoagulant like EDTA or heparin is added before the removal of blood cells.

Several studies have examined the proteomic differences between plasma and serum [6]. In the newly emerging field of metabolomics [7]–[10], there were only a few recent studies related to this subject (e.g. comparing different biofluids [11] or comparing plasma and serum from animal blood [12]). Moreover, two studies using small samples of around 15 human participants addressed this issue with conflicting results. Teahan et al. [13] reported minimal differences between the two matrices while Liu et al. [14] observed changes ranging from 0.03 to 18-fold. Here, we performed a targeted metabolomics study of 163 metabolites to compare plasma and serum samples from 377 individuals. The results showed a good reproducibility of metabolite concentrations in both plasma and serum, although somewhat better in plasma. There was also a clear discrimination between the metabolite profiles of plasma and serum. Metabolite concentrations were generally higher in serum, yet still highly correlated between the two matrices. Furthermore, serum revealed more potential biomarkers than plasma when comparing different phenotypes.

## Results

We analyzed the concentrations of 122 metabolites in both EDTA plasma and serum collected from 377 German participants of the KORA (Cooperative Health Research in the Region of Augsburg) F3 study [15], [16]. These plasma and serum samples were measured separately in 10 plates. In order to reduce potential bias and authenticate our findings, measurements on samples from 83 randomly chosen KORA F3 participants were repeated in two further plates, this time including both the plasma and the corresponding serum sample from each person in the same plate and all these samples were randomly distributed.

### Good reproducibility in serum and better in plasma

**Figure 1:**
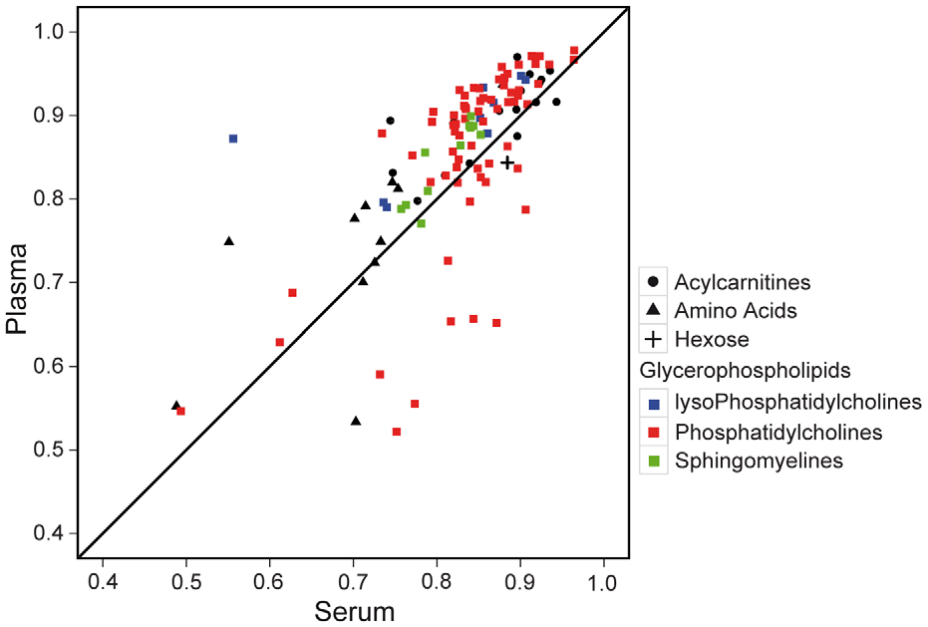
Correlation between repeated measurements of plasma and serum metabolites. Correlation coefficients (r) between repeated measurements of metabolite concentrations were plotted. r values in serum are plotted against r values in plasma. Shapes represent different groups of metabolites: Acylcarnitines (•), Amino acids (▴), Hexose (+), and Glycerophospholipid (▪). Colors represent different subgroups of glycerophospholipids: lysoPhosphatidylcholine (blue), Phosphatidylcholine (red), and Sphingomyeline (green).

Both plasma and serum displayed good stability in the metabolite concentrations we measured. The metabolite concentrations from the repeated measurements on the 83 samples showed a high correlation between the first and the second measurements (Figure 1) with mean correlation coefficients (*r*) of all 122 metabolites being 0.83 and 0.80 for plasma and serum, respectively. Thus, reproducibility was significantly better for plasma than for serum (*p* = 0.01, paired t-test), although the absolute differences in *r* were small.

### High correlation between plasma and serum metabolite concentrations and higher concentrations in serum

**Figure 2:**
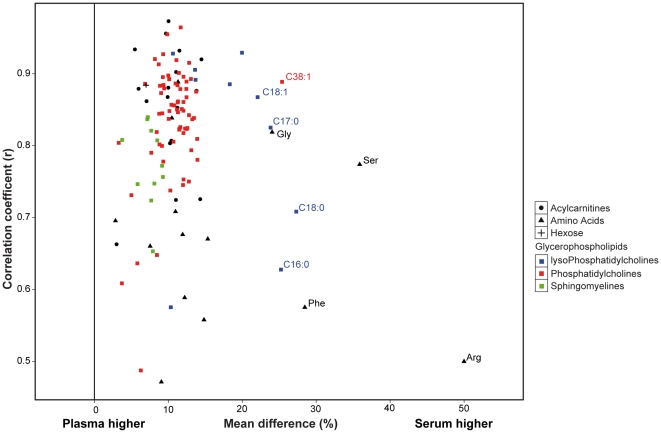
Relative concentration differences and correlation coefficients between plasma and serum for individual metabolites. The X-axis indicates the mean value of the relative concentration difference. Shapes represent different groups of metabolites: Acylcarnitines (•), Amino acids (▴), Hexose (+), and Glycerophospholipid (▪). Colors represent different subgroups of glycerophospholipids: lysoPhosphatidylcholine (blue), Phosphatidylcholine (red), and Sphingomyeline (green). Metabolite names are indicated for metabolites with a mean relative concentration difference larger than 15%. (A) 199 original individuals (B) one plate of repeated measurements from 44 individuals.

**Figure 3:**
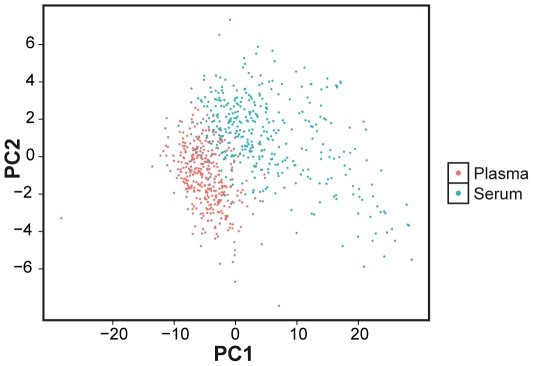
Separation of plasma and serum metabolite profiles. Results of the partial least squares (PLS) analysis. Scores of the first two PLS components were plotted against each other. Each point indicates either a plasma (red) or serum (blue) sample.

Altogether, plasma and serum samples from 83 individuals were measured in the same plates. Results showed that metabolite concentrations were generally higher in serum than in plasma (Figure 2). Out of 122 metabolites, 104 (85%) were significantly higher in serum and the average value of the relative difference over all metabolites was 11.7% higher in serum. A partial least squares (PLS) analysis of 377 KORA individuals also demonstrated that plasma samples were clearly separated from serum samples (Figure 3). In addition, we observed an overall high correlation (mean *r* = 0.81±0.1) between the values in the two matrices, indicating that differences of metabolite concentrations between both matrices are due to systematic changes across all individuals. This is especially true for most acylcarnitines (mean *r* = 0.86±0.09) and glycerophospholipids (mean *r* = 0.82±0.09). However, for amino acids, the correlation between the two matrices was significantly lower (mean *r* = 0.67±0.13) compared to all the metabolites (*p* = 0.004, t-test) (Figure 2).

Among the metabolites with significantly higher concentrations in serum, 9 metabolites had relative concentration differences greater than 20% (Figure 2). Arginine had the highest concentration difference, displaying a nearly 50% higher concentration in serum with a lower correlation (*r* = 0.50) between the two matrices, while diacyl-phosphatidylcholine C38∶1, which was 26% higher in serum than in plasma, still kept a good correlation (*r* = 0.88). Four lyso-phosphatidylcholines (C16∶0, C17∶0, C18∶0, C18∶1) and three other amino acids (serine, phenylalanine, glycine) were also found to have more than 20% higher concentrations in serum.

### Higher sensitivity in serum

We also noticed that serum provided higher sensitivity than plasma, when average metabolite concentrations were compared between subjects with different phenotypes. For example, 40 metabolites in serum were identified to have a significantly different mean concentration in type 2 diabetes (T2D) patients vs. non-T2D individuals, while plasma only revealed 25. Similar results were also observed when comparing male against female individuals, as well as when comparing smokers against non-smokers, serum always containing larger number of significantly different metabolites (Table 1). Furthermore, for each of the three phenotypes, all significantly different metabolites that were identified using plasma were among those identified using serum. The metabolites that failed to be identified in plasma were, nevertheless, close to the borderline of significance.

**Table 1:** Numbers of significant different metabolite in plasma and serum.

	Plasma (n = 377)	Serum (n = 377)
T2D vs. non-T2D (51 vs. 326)	25	40
Male vs. Female (197 vs. 180)	62	69
Smoker vs. non-smoker (45 vs. 332)	4	6

All three analyses use both plasma and serum from the same individuals. In each analysis, two different phenotypes were compared using a Wilcoxon test with the Bonferroni correction. The numbers of significantly different metabolites between phenotypes are shown in the table.

## Discussion

The present study provides a robust analysis based on a large size sample and highly reliable measurements of metabolites with stringent quality controls. The method has been proven to be in conformance with the FDA-Guideline “Guidance for Industry - Bioanalytical Method Validation (May 2001)”, which implies proof of reproducibility within a given error range. Our results give support of good reproducibility of metabolite measurements in both plasma and serum. Moreover, plasma demonstrates a better reproducibility than serum, which may result from the less complicated collecting procedure for plasma, as it does not require time to coagulate. The large sample size is not only powerful enough to detect metabolite concentration differences between the two matrices but also makes possible the further characterization of the relationship between them.

We observed that metabolite concentrations were generally higher in serum and this phenomenon may partly be explained by the volume displacement effect [17], which means that deproteinization of serum eliminates the volume fraction of proteins and distributes the remaining small molecular weight constituents in a smaller volume, thus making them more concentrated.

Concentration differences in some metabolites were similar to those observed in previous studies and some differences were related to coagulation processes. The higher arginine concentration in serum has been reported before by Teerlink et al. [18]. The release of arginine from platelets during the coagulation process might account for this difference.

Our observations that concentrations of some LPCs were higher in serum are consistent with a former study by Aoki et al. [19], who reported increased LPC concentrations, due to release of phospholipases by platelets activated by thrombin, a process that also occurs upon coagulation.

Glucose, which represents the majority of hexose, was found in an earlier study [20] to be 5% lower in plasma than in serum. A similar difference was observed for hexose in our measurements. Although the exact reason for this observation is not clear, a shift in fluid from erythrocytes to plasma caused by anticoagulants might play a role [2].

Serum demonstrated a higher sensitivity in biomarker detection. The generally higher metabolite concentrations in serum than in plasma might lead to this advantage. Metabolite measurements in both matrices are subject to a certain level of background noise, which might affect measurement accuracy, especially for metabolites with low concentrations. Thus plasma is more prone to this effect than serum, where metabolite concentrations are generally higher. It was also proposed that the lower protein content in serum might benefit small molecule analyses and improve overall sensitivity [21]. However, in our comparisons, the metabolites that differed significantly between two phenotypes in serum but not in plasma are, nevertheless, close to the significance level when plasma was used, an observation that is in agreement with the existence of high correlations between both matrices.

The high correlations between plasma and serum measurements suggest that the shift in metabolite concentrations per se does not necessarily introduce a bias in epidemiological studies, although the higher concentrations in serum may provide some advantages. In general, our data indicate that metabolite profiles from either matrix can be analyzed, as long as the same blood sample is used. However, the better reproducibility in plasma and higher sensitivity need to be taken into account, as they might influence the results for the identification of diagnostic biomarkers.

Naturally, the metabolites we measured in our experiment represent only a small part of the human blood metabolome. Accordingly, it is yet to be determined in future studies whether similar observations can be made for other metabolites.

## Materials and Methods

We obtained written informed consent from all participants and approval from the local ethical committee.

KORA is a population-based research platform with subsequent follow-up studies in the fields of epidemiology, health economics and health care research [15]. It is based on interviews in combination with medical and laboratory examinations, as well as the collection of biological samples.

Plasma and serum from 377 individuals (180 female, 197 male, age range from 51 to 84 years) from the population based cohort KORA F3 [22] were used in this study. For each participant, fasting blood was simultaneously drawn into serum and EDTA plasma gel tubes between 8 and 10 am. Plasma tubes were shaken gently and thoroughly for 15 minutes followed by centrifugation at 2750 g for 15 minutes at 15°C. In the meantime, serum tubes were gently inverted twice followed by 45 minutes resting at room temperature to obtain complete coagulation, before performing the same centrifugation process as for plasma. All samples were stored at -80°C until the metabolic analyses.

In total, 26 quantified and 137 semi-quantified (due to the lack of standards) metabolites were measured in 377 plasma and serum samples using a commercially available metabolomics kit (Absolute*IDQ*™ kit p150, Biocrates Life Sciences AG, Innsbruck, Austria) based on FIA-MS. The assay procedures and the full biochemical names have been described in more detail in our previous work [8], [23]–[25] and the analytical details have been reviewed and compared with other analytical technology [26].

The first measurement of 377 plasma and serum samples took place in two separate batches, with a three months time interval in between. Each batch contained 5 plates. To ensure the detection of true effects, measurements on samples from 83 randomly chosen KORA F3 participants from all 5 plates were repeated on two additional plates (39 and 44 samples, respectively), with both the plasma and the corresponding serum samples from the same person being measured in the same plates and samples randomly distributed in the plates.

To control data quality, a metabolite had to meet three criteria: (I) the average value of the coefficient of variance of the three quality control samples (representing human plasma samples provided by the manufacturer in each kit plate) should be smaller than 0.25; (II) the mean concentration of the metabolite over all samples should be above 0.1 µM or the metabolite concentration should be above the limit of detection (LOD) for 90% of the samples. The LODs were set to three times the values of zero samples (e.g. extraction solution or PBS with internal standards); (III) the correlation coefficient between the 83 repeated measurements in either specimen must exceed 0.5. Altogether, 25 quantified and 97 semi-quantified metabolites passed both criteria (Table S1).

In order to detect real differences between plasma and serum rather than the differences between plates, a plate correction factor was calculated for each plate using the plate containing re-measured samples from 39 individuals as a basis: the geometric mean was taken for each pair of metabolite concentrations determined from the repeated measurements on 39 plasma and 39 serum samples and divided by the concentration determined in the respective plate, yielding 78 ratios per metabolite. Subsequently, for each of the 10 original plates, the geometric mean of all the ratios was calculated and used as plate-specific correction factors.

Partial least squares (PLS) was performed using scaled metabolite concentrations to investigate a possible separation of metabolite profiles between plasma and serum samples. For each metabolite, the Pearson's correlation coefficient (r) was calculated, and a paired Wilcoxon test was performed to compare metabolite concentrations in plasma vs. serum. In addition, to calculate the relative concentration differences, we divided the absolute concentration difference (serum minus plasma) of each metabolite by its average concentration over the two matrices.

Three analyses were performed to compare metabolite concentrations in (I) diabetes patients vs. normal individuals, (II) male vs. female individuals and (III) smokers vs. non-smokers using both plasma and serum samples. The group of diabetes patients included self-reported cases and subjects reported to take diabetic medications. In all three analyses, we used a Wilcoxon test to find metabolites that are significantly different between the respective groups and the same procedures were applied to both plasma and serum samples. In all the three analyses mentioned above, different phenotypes were randomly distributed among plates.

All calculations were performed under the R statistical environment (http://www.r-project.org/).

## Supporting Information

Table S1 **Summary of metabolites in plasma and serum samples.**
(XLS)
